# Fourier-Sparsity Integrated Method for Complex Target ISAR Imagery

**DOI:** 10.3390/s150202723

**Published:** 2015-01-26

**Authors:** Xunzhang Gao, Zhen Liu, Haowen Chen, Xiang Li

**Affiliations:** College of Electronic Science and Engineering, National University of Defense Technology, Changsha 410073, China; E-Mails: zhen_liu@nudt.edu.cn (Z.L.); chenhw@nudt.edu.cn (H.C.); lixiang01@vip.sina.com (X.L.)

**Keywords:** inverse synthetic aperture radar, sparse recovery, sparsity-driven, range compression, range cell migration, complex targets

## Abstract

In existing sparsity-driven inverse synthetic aperture radar (ISAR) imaging framework a sparse recovery (SR) algorithm is usually applied to azimuth compression to achieve high resolution in the cross-range direction. For range compression, however, direct application of an SR algorithm is not very effective because the scattering centers resolved in the high resolution range profiles at different view angles always exhibit irregular range cell migration (RCM), especially for complex targets, which will blur the ISAR image. To alleviate the sparse recovery-induced RCM in range compression, a sparsity-driven framework for ISAR imaging named Fourier-sparsity integrated (FSI) method is proposed in this paper, which can simultaneously achieve better focusing performance in both the range and cross-range domains. Experiments using simulated data and real data demonstrate the superiority of our proposed framework over existing sparsity-driven methods and range-Doppler methods.

## 1. Introduction

In recent years, inverse synthetic aperture radar (ISAR) imaging techniques exploiting sparse recovery (SR) algorithm have attracted growing attention due to their intrinsic advantage of exceeding the resolution limitation of the conventional Fourier-transform based range-Doppler (RD) method [1–4]. In ISAR imaging scenarios, the reflectivity function of the air scenes is typically almost zero out of the target boundaries and the radar image of the target representing the spatial distribution of limited scattering centers occupies a minority of the image pixels [5]. Thus scattering centers are expected to be sparse in the ISAR image and ISAR imaging can be formulated as a sparse recovery problem assuming that the translational motion has been compensated and there is no range cell migration (RCM) during the radar coherent processing interval (CPI). In some ISAR imaging literatures, RCM is also called migration through resolution cells (MTRC).

Generally, any ISAR imaging process consists of two consecutive steps: range compression and cross-range compression and in both of these procedures a SR algorithm could be exploited. In range compression some SR-based imaging algorithms have been explored to reduce sampling frequency, sampling time as well as measured data size [3,6,7]. In cross-range compression many sparsity-driven ISAR imaging methods have been proposed to improve autofocus quality and cross-range resolution [8–12]. Assuming that the translational motion has been already compensated, the ISAR image could also be derived via two-dimensional joint SR algorithms [13–17]. In particular, by building a parametric dictionary matrix the authors of [14–16] proposed a novel sparsity-driven imaging framework which could simultaneously compensate RCM caused by the rotational motions.

According to the principle of the SR algorithm, the ISAR image of the target could be reconstructed perfectly provided that all the scattering centers lie on the center of grid points in the imaging region. However no matter how small the size of the grid cell is, the actual scattering centers will not always lie on the center of the grid points. In other words, grid misalignment usually arises in sparse recovery. When a scattering center lies between two cells of a discretely-resolved range-Doppler plane, it will spill non-zero values into all cells with spillage amplitude following a Dirichlet kernel [18]. Therefore some fake scattering centers appear in adjacent range cells near the true location. During range compression, the spillage will cause irregular RCM and then blur the ISAR image. Because of its irregular migration way, this grid misalignment-induced RCM is impossible to remove with the conventional RCM compensation methods such as the keystone transform, which was developed for compensating the RCM caused by rotational motion [19]. Similarly, the sparsity-driven imaging framework proposed in [14–16] could not deal with this irregular RCM. It is for this reason that direct application of SR algorithms in both range compression and cross-range compression simultaneously cannot derive favorable ISAR imaging results as expected. To address this issue, this paper firstly elaborates the mechanism of the RCM induced by an SR algorithm and then proposes a sparsity-driven framework for ISAR imaging named the Fourier-sparsity integrated (FSI) method, which can effectively avoid producing irregular RCM and greatly improve the focusing performance in both the range and cross-range domains.

This paper is organized as follows: Section 2 briefly introduces the principals of range and cross-range compression with sparsity-driven methods and illustrates the involved RCM phenomenon. To alleviate the influence of SR-induced RCM on ISAR images, a novel FSI method is proposed in Section 3. In Section 4 the performance comparison between our framework and existing methods is presented by using simulated data and real data. Finally some conclusions are drawn in the last section.

## 2. RCM Induced by Sparse Recovery Algorithm

Assuming that the translational motion has been fully compensated, ISAR imaging may be considered as a turntable model imaging within a 2-dimensional plane which consists of two steps: range and cross-range compression. Theoretically, both of these steps can be implemented via SR algorithms.

### 2.1. Range Compression via a SR Algorithm

**Figure 1.**
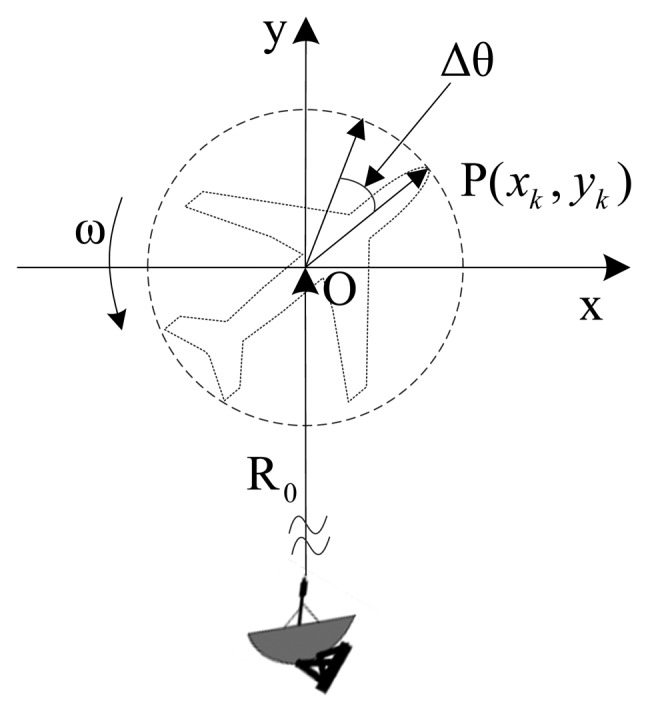
Geometry of turntable model imagery.

The geometry of turntable model imaging is shown in Figure 1. We assume that during a short CPI the rotation angle is very small and the Doppler shifts nearly remain constant. The instantaneous distance at time instant *t* from the scattering center to the radar can be approximated as [10]:

(1) $$R_{k}(t)=R_{0}+r_{k}\approx R_{0}+y_{k}+x_{k}\omega t$$

where ω stands for the rotation rate. Suppose that the radar transmits the linear frequency modulated (LFM) signal:

(2) $$s_{t}\left(\tau ,t_{m}\right)=\mathrm{rect}\left(\tau /T_{r}\right)\exp\left[j2\pi \left(f_{c}t+{\gamma \tau }^{2}/2\right)\right]$$

where τ = *t*–*t_m_* is the fast time; *t_m_* = *mT_r_* is the slow time, *T_r_* denotes the pulse repetition interval (PRI) and *m* = 0,1, …*M*−1 is the transmitted pulse number index; rect(·) denotes the unit rectangle function; *f_c_* and γ denote the carrier frequency and the chirp rate, respectively.

Suppose that the target contains *K* scattering centers, *σ_k_* is the *k* th scattering intensity, *R_k_*(*t_m_*) is its range to radar, corresponding time-delay τ*_km_* = 2*R_k_*(*t_m_*)/*c*, *c* is the speed of the light. The complex envelope of the echo signal can be written as:

(3) $$\begin{array}{ll} s_{r}(\tau ,t_{m}) & =\overset{K}{\underset{k=1}{\text{∑}}}\sigma_{k}\mathrm{rect}\left(\left(\tau -\tau_{km}\right)/T_{r}\right)\\ & \times \exp\left\{j2\pi \left[f_{c}\left(t-\tau_{km}\right)+\gamma {\left(\tau -\tau_{km}\right)}^{2}/2\right]\right\} \end{array}$$

Assume that reference range for dechirping is denoted by *R_ref_*(*t_m_*), and the corresponding time-delay by τ_0_*_m_* = 2*R_ref_*(*t_m_*)/*c*. The reference signal is given by:

(4) $$\begin{array}{ll} s_{ref}\left(\tau ,t_{m}\right) & =\mathrm{rect}\left(\left(\tau -\tau_{0m}\right)/T_{ref}\right)\\ & \times \exp\left\{j2\pi \left[f_{c}\left(t-\tau_{0m}\right)+\gamma {\left(\tau -\tau_{0m}\right)}^{2}/2\right]\right\} \end{array}$$

where *T_ref_* is the reference pulse width (*T_ref_* > *T_r_*). Omitting the rectangle function and assuming that the residual video phase (RVP) has been removed, the output signal after dechirping can be formulated as:

(5) $$s\left(\tau ,t_{m}\right)=\overset{K}{\underset{k=1}{\text{∑}}}\sigma_{k}\exp\left\{-j4\pi r_{k}(f_{c}+\gamma \tau )/c\right\}$$

Let τ = *n*·Δτ, *n* = 0,1,⋯*N*−1. Neglecting the constant phase term, the discretized echo at some aspect can be expressed by:

(6) $$s(n)=\overset{K}{\underset{k=1}{\text{∑}}}\sigma_{k}\exp\left\{-j2{\pi \omega }_{k}n\right\}$$

where ω*_k_* = 2*r_k_*γΔτ/*c* and ω*_k_* ∈ [0,1). Let ω*_k_* = *p*/*P*, *p* = 0,1,⋯,*P*−1. The sparse representation formula of the echo data can therefore be given as:

(7) $$s=\Theta u+{\text{n}}_{r}$$

where **s** ∈ ℂ*^N^*^×1^ denotes the echo vector, **Θ** ∈ ℂ*^N^*^×1^(*N* < *P*) is a partial Fourier matrix with elements **Θ**[*n*,*p*] = exp(−*j*2π*np* / *P*). **u** ∈ ℂ*^P^*^×1^ is the complex range profile whose *K*(*K* < *P*) nonzero components correspond to the synthetic complex amplitudes of the range cells and vector **n***_r_* represents the additive noise. The complex amplitude vector in Equation (7) can be estimated by introducing the following constrained sparsity-enforcing objective function:

$$\begin{array}{cc} \hat{\text{u}}=\arg\underset{\text{u}}{\min}{\left\|\text{u}\right\|}_{0} & \text{subject to}\;{\left\|\text{s}-\Theta \text{u}\right\|}_{2}\leq \varepsilon \end{array}$$

where ε is the noise level satisfying ε =‖**n***_r_*‖_2_, ‖·‖*_p_* denotes *l_p_* norm and min (·) denotes the minimization. According to the compressed sensing theory, when matrix **Θ** satisfies the restricted isometry property (RIP) or incoherent condition, we can reconstruct the sparse signal vector **u** in Equation (7) via SR method with high probability [20].

**Figure 2.**
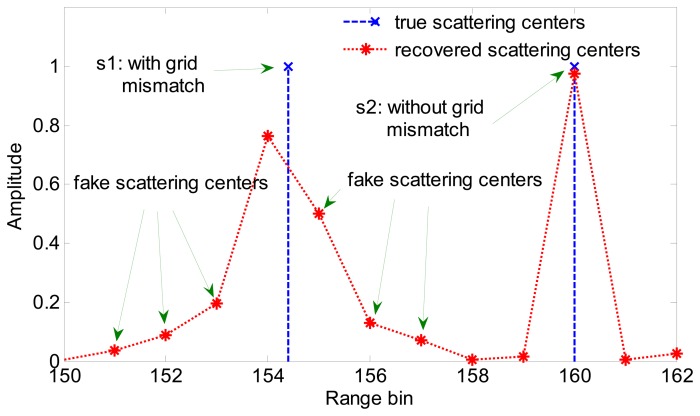
Reconstructed result via SR algorithm of two scattering centers.

According to [18] no matter how small the size of the grid cell is, grid misalignment usually arises in sparse recovery. The scattering center that lies between two cells of a discretely-resolved range-Doppler plane will spill non-zero values into all cells with spillage amplitude following a Dirichlet kernel. Figure 2 depicts the reconstructed result of a target consisting of two scattering centers near to each other. Considering that ISAR imaging processing is always performed in the complex-valued domain, an effective and efficient algorithm named SL0 [21] is employed for sparse recovery. In this simulation, the scattering center s1 is with grid mismatch and s2 is without grid mismatch. That is to say, s1 locates between two grid points and s2 lies on a gird point. As can be seen clearly from Figure 2, s2 is precisely recovered but for s1 some fake scattering centers appear in adjacent range cells near the true location. In Figure 2, the true locations and the recovered locations are indicated with blue crosses and red asterisks, respectively.

**Figure 3.**
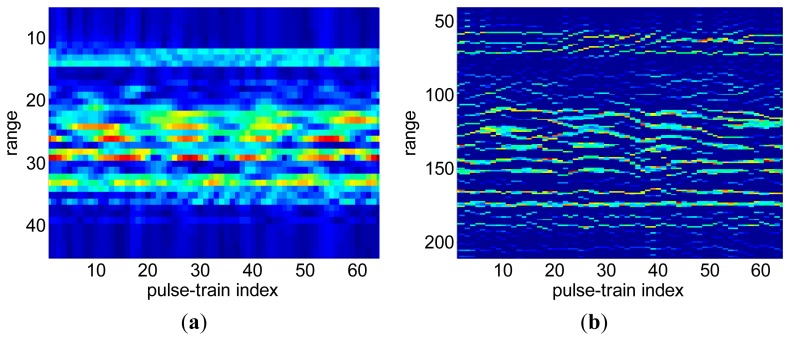

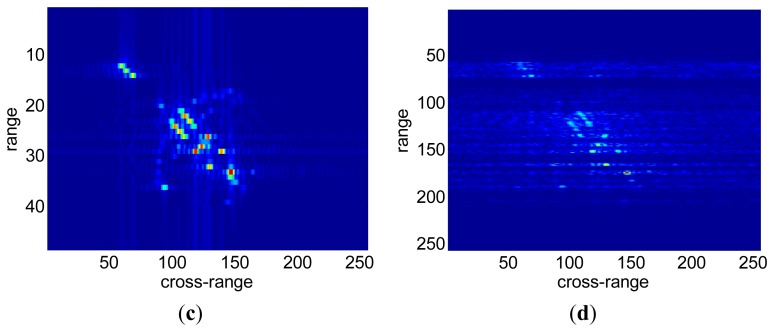
(**a**) Range profiles via IFFT method (64 samples for each echo pulse); (**b**) Range profiles with irregular RCM via SR algorithm (256 grid points); (**c**) ISAR image corresponding to the range profiles in 3a; (**d**) ISAR image corresponding to range profiles in 3b.

For complex targets, scattering centers with minor range difference often do exist. After range compression via the SR method, these scattering centers will be resolved into different range cells. Due to the varying locations of scattering centers at different view angles as well as the grid mismatch, scattering centers would not always be located in the same range cell for all range profiles. In other words, when an SR algorithm is applied for range compression, irregular RCM and fake scattering centers always occur, as can be seen from Figure 3b. This irregular RCM results in the ribbon like defocusing in the cross-range direction as shown in Figure 3d. On the contrary, these adjacent scattering centers cannot be resolved by low resolution methods such as IFFT-based methods due to the resolution limitations. Hence, its RCM effect is not serious during range compression, as illustrated in Figure 3a. Accordingly the cross-range defocus of the ISAR image in Figure 3c is not as serious as in Figure 3d.

By comparing the imaging results, it can be seen that there seems to be a conflict between range resolution and RCM during range compression procedures. This phenomenon motivates further studies described below.

### 2.2. Cross-Range Compression via SR Algorithm

After range compression, the received signal becomes [9,10]:

(8) $$\begin{array}{ll} S\left(\tau ,t_{m}\right) & =\overset{K}{\underset{k=1}{\text{∑}}}\sigma_{k}\mathrm{rect}\left(\frac{t_{m}}{T_{a}}\right)\mathrm{sinc}\left[T_{p}\gamma \left(\tau -\frac{2\left(R_{0}+y_{k}\right)}{c}\right)\right]\\ & \times \exp\left\{-j4\pi R_{k}(t_{m})/\lambda \right\} \end{array}$$

where *T_a_* and *T_p_* denote the observation duration and pulse width respectively; λ = *c*/*f_c_* is the wavelength. Substituting Equation (1) into Equation (8) and assuming that the range cell corresponding to τ = 2(*R*_0_ + *y_k_*)/*c* contains *L* ≤ *K* scattering centers with different cross-range locations, the signal in the range cell can be simplified by neglecting the constant phase term as [10,11]:

(9) $$S(t_{m})=\overset{L}{\underset{l=1}{\text{∑}}}\sigma_{l}^{\prime }\mathrm{rect}\left(\frac{t_{m}}{T_{a}}\right)\exp\left(-j4\pi x_{l}\omega t/\lambda \right)$$

where $\sigma_{l}^{\prime }$ denotes the *l* th scattering centers' intensity. Substituting *t_m_* = *mT_r_* into Equation (9), the discretized signal corresponding to a range cell in Equation (9) can be reformatted as:

(10) $$S(m)=\overset{L}{\underset{l=1}{\text{∑}}}\sigma_{l}^{\prime }\exp\left(-j2\pi \varpi_{l}m\right)$$

where ϖ*_l_* = 2*x_l_*ω*T_r_*/λ and ϖ*_l_* ∈ [0,1). Let ϖ*_l_* = *q*/*Q* and considering the synthetic additive noise in the range cell, Equation (10) can be rewritten as:

(11) $$\text{S}=\Phi \text{a}+{\text{n}}_{c}$$

where **S** ∈ ℂ*^M^*^×1^ denotes the signal vector, **Φ** ∈ ℂ*^M^*^×^*^Q^* is a partial Fourier matrix with element **Φ**[*m*,*q*] = exp(−*j*2π*mq* /*Q*). **a** ∈ ℂ*^Q^*^×1^ is the vector in a range cell, whose nonzero components correspond to the scattering center's intensity of the cross-range cells and vector **n***_c_* represents the additive noise. Consequently, cross-range compression can also be implemented via an SR algorithm.

## 3. The Proposed Sparsity-Driven ISAR Imaging Framework

Aiming to overcome the conflict between range resolution and RCM described in Section 2.1, we propose a novel framework for complex target ISAR imaging named FSI method by combining the advantages of the RD method and the sparse representation technique. The key idea of the proposed FSI method is to complete range compression via a low resolution method firstly, so as to keep the near scatters in the same resolution range cell during the CPI, then resolve the scatters in each range cell along the cross-range direction via a high resolution method, thus the coarse ISAR image can be derived. Afterwards the echo data of each cross-range cell are synthesized by FFT operation on the coarse image data along range direction. Lastly the scatters in each cross range cell can be finely resolved by applying SR method for echo data of all cross-range cells. The above process skillfully avoids the RCM induced by the SR based range compression.

**Figure 4.**
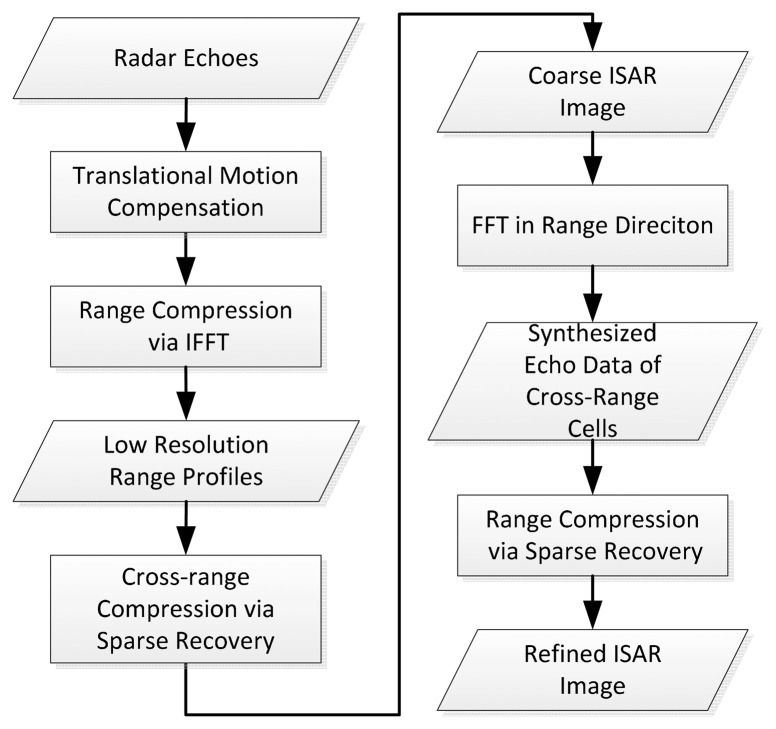
Block diagram of the proposed FSI method.

The main steps of the FSI method are depicted in Figure 4 and summarized as follows:

(1) For a moving target, the discrete radar echo data *s*(τ,*t_m_*) given in Equation (5) are collected after translational motion compensation.
(2) For each echo pulse *s*(τ,*t_m_*), and complete range compression using the IFFT algorithm, then *M* low resolution range profiles *S*(τ,*t_m_*) are derived, and each range profile includes *N* range cells.
(3) For each range cell of the range profiles, scatters can be resolved along cross-range direction via SR algorithm described in Section 2.2, thus the coarse ISAR image matrix **A***_c_* consisting of *N*×*Q* grids (*Q*>*M*) can be obtained, and each row of **A***_c_* is the optimization solution of vector **a** defined in Equation (11) via the SR method.
(4) Synthesize the echo data of each cross-range cell *S_s_* ∈ ℂ*^N^*^×1^ by implement FFT operation on each column of the coarse ISAR image **A***_c_*.
(5) Applying the SR algorithm described in Section 2.1 to the synthesized echo data *S_s_* ∈ ℂ*^N^*^×1^, the vector **u** ∈ ℂ*^P^*^×1^ representing the distribution of the scattering centers of a cross-range cell can be reconstructed, then the refined complex ISAR image **A***_r_* ∈ ℂ*^P^*^×^*^Q^* can be reformulated.

As the method consists of one FFT-based range compression, one FFT-based date synthesizing operation and two SR-based cross-range compressions, its computational costs is about double that of the existing IFFT-SR method. The computation load mainly depends on the employed SR algorithm.

## 4. Experimental Section

In this section, the performances of our proposed FSI method are evaluated by using simulated data and real data, and comparisons with other existing methods are also presented.

### 4.1. Simulated Data Experiments

**Figure 5.**
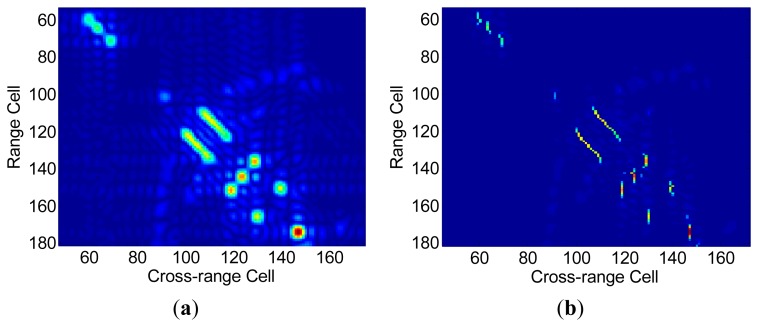

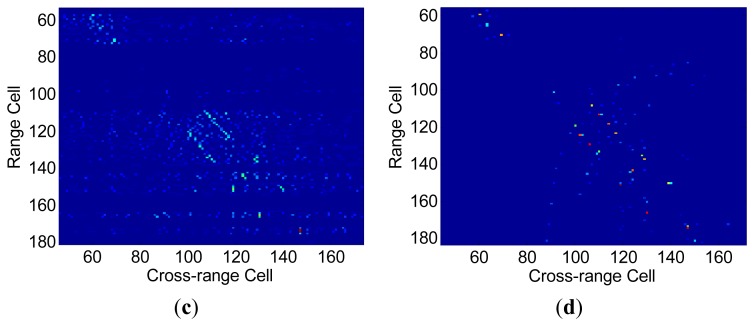
(**a**) ISAR image via IFFT-IFFT method; (**b**) ISAR image via existing IFFT-SR method; (**c**) ISAR image via SR-SR method; (**d**) ISAR image via the proposed FSI method.

Here we utilize the widely used simulated B-727 data provided online by the U.S. Naval Research Laboratory to evaluate the performance quantitatively and qualitatively The stepped frequency radar operates at 9 GHz and has a bandwidth of 150 MHz. There are 256 successive pulse-trains and for each pulse-train 64 complex range samples are saved. Herein the B-727 data for simulation is under high SNR and motion compensation has been applied. To demonstrate the high resolution ability of the SR algorithm, only 64 successive pulse-trains and 48 complex range samples for each pulse are utilized. In all experiments the SL0 algorithm is employed for sparse recovery algorithm. As shown in Figure 5d, it can be observed that the strong scattering centers of the simulated B-727 are well focused and resolved with high resolution both in range and cross-range domain by FSI method. For comparison, the conventional Fourier-based (IFFT-IFFT method) ISAR image is shown in Figure 5a. One can see that the dominant scatters are blurred due to the narrow bandwidth and short coherent time.

Figure 5b is obtained via the existing sparsity-driven IFFT-SR method, where range compression is implemented via the IFFT method with padded zeros and cross-range compression is implemented via an SR algorithm. However, the scatters are not well resolved although the cross-range resolution is improved by SR method. Figure 5c shows the ISAR image obtained via the SR-SR method, where both range compression and cross-range compression are implemented via a SR method. In Figure 5c, severe defocusing in the cross-range domain appears due to the SR-induced irregular RCM. In the field of ISAR imaging, image entropy and image contrast are often considered as two ways of measuring the focus of an ISAR image [22–25]. In our case image contrast (IC) [22] and image entropy (IE) [23] are defined as below, respectively:

(12) $$\begin{array}{ll} \text{IC} & =\frac{\sqrt{\mathrm{Ave}\left\{{\left[{\text{I}}^{2}-\mathrm{Ave}\{{\text{I}}^{2}\}\right]}^{2}\right\}}}{\mathrm{Ave}\{{\text{I}}^{2}\}}\\ \text{IE} & =-\mathrm{Sum}\left\{\frac{{\text{I}}^{2}}{\mathrm{Sum}\{{\text{I}}^{2}\}}\ln\left(\frac{{\text{I}}^{2}}{\mathrm{Sum}\{{\text{I}}^{2}\}}\right)\right\} \end{array}$$

where I^2^ ∈ ℝ*^P^*^×^*^Q^* denotes the intensity matrix of the ISAR image and its (*m*,*n*)*^th^* element equals to the square of the corresponding image pixel value, *i.e.*, I^2^ (m,n) = [I(m,n)]^2^. *Ave*{•} is the spatial mean operator and Sum{•} represents spatial summation operator. For a real matrix I ∈ ℝ*^P^*^×^*^Q^*_:_

(13) $$\begin{array}{cc} \mathrm{Sum}\{\text{I}\}=\overset{P}{\underset{m=1}{\text{∑}}}\overset{Q}{\underset{n=1}{\text{∑}}}\text{I}(\text{m},\text{n}), & \mathrm{Ave}\{\text{I}\}=\frac{1}{PQ}\mathrm{Sum}\{\text{I}\} \end{array}$$

Both image contrast and image entropy can give a measure of the difference in the intensity between the scatters and the backgrounds. For well-focused ISAR images a high contrast value and a low entropy value are expected. Instead, the amplitude of an unfocused image is concentrated around its mean value, thus the contrast value is low and the entropy value is high.

**Figure 6.**
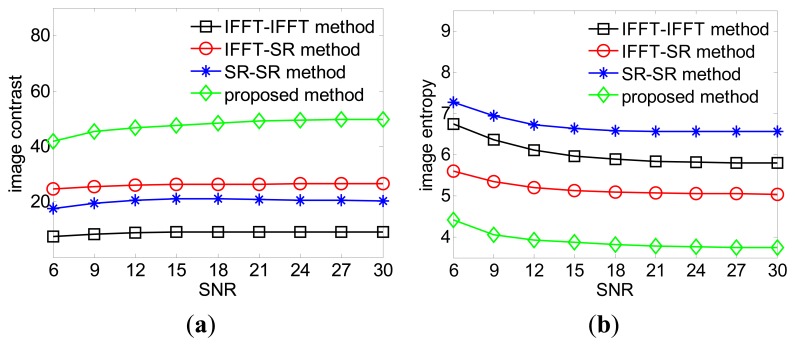
(**a**) Focusing performance of different methods: IC *versus* SNR; (**b**) Focusing performance of different methods: IE *versus* SNR.

More experiments are performed at different signal noise ratios (SNRs) by adding appropriate amounts of zero-mean, white Gaussian noise to the simulated raw data. For each SNR, the IC values and IE values are averaged over 100 simulations. It is easy to observe that our proposed method achieves the largest IC value and smallest IE value in each SNR scenario. According to Figures 5 and 6, it is reasonable to conclude that our proposed FSI method can inherently achieve better focusing performance than the existing ISAR imaging framework.

### 4.2. Real Data Experiments

**Figure 7.**
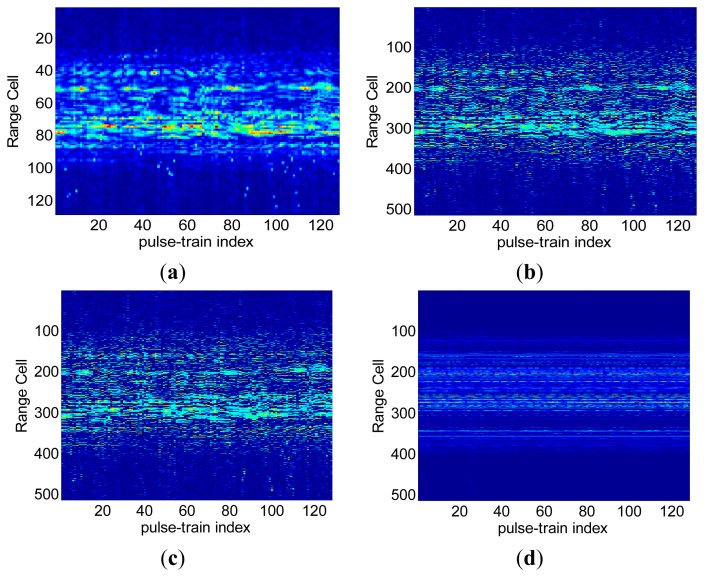
(**a**) Range profiles via IFFT method; (**b**) Range profiles via SR method; (**c**) Range profiles via SR method with range alignment; (**d**) Range profiles by FFT transform on FSI method formulated ISAR image.

**Figure 8.**
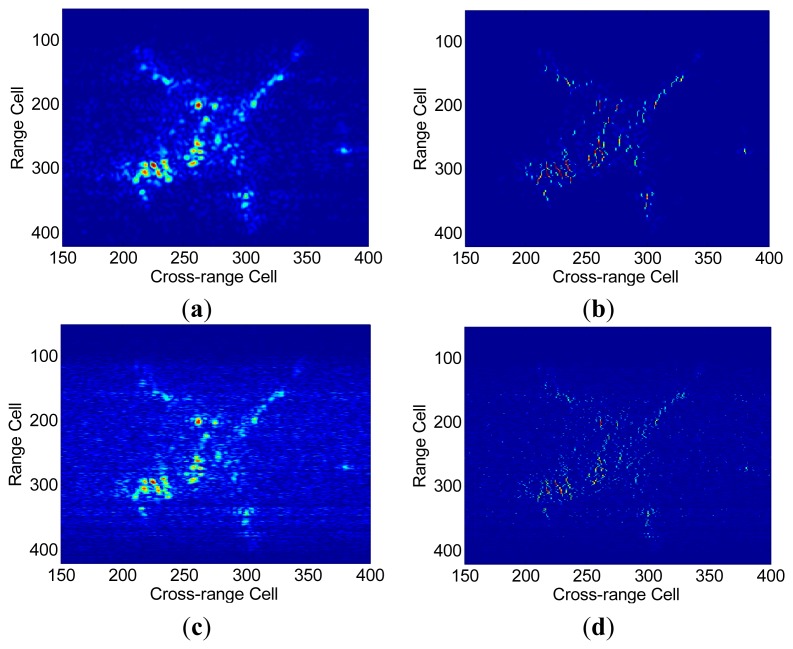

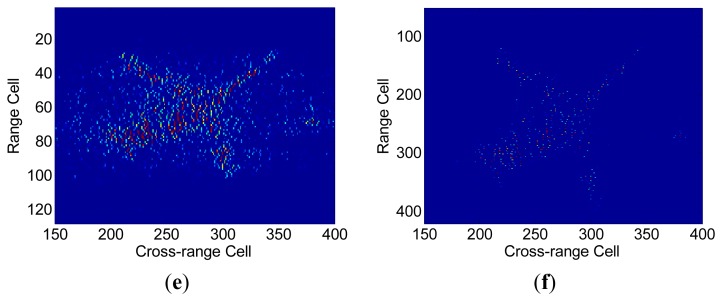
(**a**) ISAR image via IFFT-IFFT method; (**b**) ISAR image via existing IFFT-SR method; (**c**) ISAR image via SR-IFFT method; (**d**) ISAR image via SR-SR method; (**e**) Coarse ISAR image via FSI method; (**f**) Refined ISAR image via FSI method.

The proposed FSI method is also validated with a set of real data of theYak-42 plane. This set of data is measured by a ground-based experimental radar which works at C-band (central frequency 5.52 GHz) and transmits chirp signals with bandwidth = 400 MHz, corresponding to a range resolution of 0.375 m. The pulse repetition frequency (PRF) of the data set is 100 Hz. 128 complex samples are collected for each pulse and 128 pulses within dwell time [−0.64 s, 0.64 s] are used in this experiment. The translational motion has been compensated with existing method for this data set. In the SR imaging procedure, a partial Fourier dictionary with 512 columns and 128 rows is utilized. Figure 7a shows the range profiles via conventional IFFT method. Generally speaking, scatters in each range cell stay in place consistently during the CPI. By contrast, in Figure 7b a severe range migration of the scatters and the discontinuities between range profiles can be observed, which illustrates the appearance of RCM during SR-based range compression. To show the irregularity of this RCM, range alignment via a traditional correlation method [26] is performed on these range profiles, but as shown in Figure 7c, the disorder between range profiles is not alleviated at all, which implies that SR- induced RCM is different from the rotation-induced RCM and cannot be removed by existing compensation algorithms. Range profiles in Figure 7d are inverted from the FSI method formulated ISAR image by FFT transform. It can be seen that the range profiles are neatly aligned and no apparent RCM can be observed, which indicates that the proposed FSI method can avoid irregular RCM in the imaging procedure very effectively. In Figure 8a–d, ISAR images are presented to qualitatively evaluate the performance of our proposed FSI method. Using the complex range profiles in Figure 7a, we derive the images in Figure 8a,b via the IFFT method and SR method, respectively. As expected, the SR method obtains a better quality image with high cross-range resolution than the IFFT (R-D) method. Similarly, images in Figure 8c,d are obtained via the IFFT and SR method using the complex range profiles in Figure 7b, respectively. Due to the SR-induced RCM, both of these images are less-focused in comparison with images in Figure 8a,b.

The coarse ISAR image and refined ISAR image via the FSI method are presented in Figure 8e,f, respectively. It can be observed clearly that the scatters in Figure 8f are well resolved both in range and cross range domain with low side-lobes and the plane profile is clearly recognized. The comparison of the imaging results manifests that our proposed method achieves the best focal quality among these ISAR imaging approaches.

In Table 1, image contrast and image entropy are given to compare the quality of the ISAR images quantitatively. Apparently the image via FSI method obtains the largest image contrast value and the smallest image entropy value, which suggests the better performance of our proposed FSI method over other existing methods in real applications.

## 5. Conclusions

In this paper the irregular RCM between profiles at different view angles during sparstity-driven ISAR imaging process is elaborated first. It is claimed that the inevitable grid mismatch of sparse recovery algorithms is the main reason that causes SR-induced RCM when performing range compression for complex targets. By first performing the cross-range focusing and then refining the range profile via an SR algorithm, a novel sparsity-driven ISAR imaging framework named Fourier-sparsity integrated (FSI) method is proposed to improve focusing performance in both the range and cross-range domains simultaneously. Experimental results using simulated data and real data demonstrate that the proposed FSI method can effectively alleviate this irregular RCM and outperform existing sparsity-driven frameworks as well as the R-D method in focal quality and side-lobe depression. However, as this approach is established on the assumption of precise motion compensation, its application in ISAR imaging for complex motion target would be of interest for future work.

## Figures and Tables

**Table 1.** ISAR image quality.

**Method**	**IFFT-IFFT**	**IFFT-SR**	**SR-IFFT**	**SR-SR**	**FSI**
IC	7.9050	24.6181	3.9159	15.9429	35.8454
IE	9.5956	7.0915	10.9801	8.6307	6.1100
